# Brain Network Analysis: Separating Cost from Topology Using Cost-Integration

**DOI:** 10.1371/journal.pone.0021570

**Published:** 2011-07-28

**Authors:** Cedric E. Ginestet, Thomas E. Nichols, Ed T. Bullmore, Andrew Simmons

**Affiliations:** 1 Department of Neuroimaging, Institute of Psychiatry, King's College London, London, United Kingdom; 2 National Institute of Health Research (NIHR) Biomedical Research Centre for Mental Health, Institute of Psychiatry, King's College London, London, United Kingdom; 3 Department of Statistics, University of Warwick, Coventry, United Kingdom; 4 Brain Mapping Unit, Department of Psychiatry, School of Clinical Medicine, University of Cambridge, Cambridge, United Kingdom; Indiana University, United States of America

## Abstract

A statistically principled way of conducting brain network analysis is still lacking. Comparison of different populations of brain networks is hard because topology is inherently dependent on wiring cost, where cost is defined as the number of edges in an unweighted graph. In this paper, we evaluate the benefits and limitations associated with using cost-integrated topological metrics. Our focus is on comparing populations of weighted undirected graphs that differ in mean association weight, using global efficiency. Our key result shows that integrating over cost is equivalent to controlling for any monotonic transformation of the weight set of a weighted graph. That is, when integrating over cost, we eliminate the differences in topology that may be due to a monotonic transformation of the weight set. Our result holds for any unweighted topological measure, and for any choice of distribution over cost levels. Cost-integration is therefore helpful in disentangling differences in cost from differences in topology. By contrast, we show that the use of the weighted version of a topological metric is generally not a valid approach to this problem. Indeed, we prove that, under weak conditions, the use of the weighted version of global efficiency is equivalent to simply comparing weighted costs. Thus, we recommend the reporting of (i) differences in weighted costs and (ii) differences in cost-integrated topological measures with respect to different distributions over the cost domain. We demonstrate the application of these techniques in a re-analysis of an fMRI working memory task. We also provide a Monte Carlo method for approximating cost-integrated topological measures. Finally, we discuss the limitations of integrating topology over cost, which may pose problems when some weights are zero, when multiplicities exist in the ranks of the weights, and when one expects subtle cost-dependent topological differences, which could be masked by cost-integration.

## Introduction

In the last decade, the biological and physical sciences have witnessed a proliferation of publications adopting a network approach to a wide range of questions. This interest in networks was originally stimulated by the seminal works of Watts et al. [1] and Barabasi et al. [2], who introduced the concepts of small-world and scale-free networks, respectively. Some of these ideas have been adopted in neuroscience at both a theoretical [3], [4] and experimental level [5]. Most of the research in this area has attempted to classify the topology of brain networks based on anatomical or functional data [6]–[8].

A question that naturally arises from such applications of graph theory is whether or not the topological properties of these brain networks are stable across different populations of subjects or across different cognitive and behavioral tasks. A common hypothesis that neuroscientists may wish to test is whether the small-world properties of a given brain network are conserved when comparing patients and healthy controls. Bassett et al. [9], for example, have studied differences in anatomical brain networks between healthy controls and patients with schizophrenia. Other authors have evaluated whether the topological properties of functional networks vary with different behavioral tasks [10]–[13]. The properties of brain network topology have also been studied at different spatial scales [14] and using different modalities, such as EEG [15], [16], and fMRI [6], [7]. There is therefore considerable interest in comparing populations of networks –which may represent different groups of subjects, several conditions of an experiment, or the use of different levels of spatial or temporal resolution. We note that such research questions are more likely to arise when subject-specific networks can be directly constructed. This has been done in the context of both functional and structural MRI [17], [18].

The possibility of conducting rigorous statistical comparison of several populations of networks, however, has been hindered by a series of methodological issues, which have not been hitherto satisfactorily resolved. When considering the question of comparing several populations of networks, two main problems arise. Firstly, we are faced with the inherent intertwining of connectivity strength (i.e. wiring cost) with network topology. Most topological metrics used to compare networks are sensitive to differences in these graphs' number of edges. Drawing comparisons on the sole basis of topology therefore requires some level of control of cost discrepancies between these network populations. Secondly, this issue is compounded by the fundamental division between weighted and unweighted graphs. The problem of disentangling differences in connectivity strength from topological differences therefore needs to be resolved in a distinct manner depending on whether weighted or unweighted graphs are being considered. The focus, in this paper, will be on weighted networks since these are more likely to be found in the biomedical sciences than their unweighted counterparts.

Historically, however, network analyses have concentrated on unweighted graphs. The application of graph theory to biological and artificial networks was originally motivated by the discrete nature of the problems of interest. Both Watts et al. [1] and Barabasi et al. [2] mainly considered binary relations between sets of elements, which readily produced adjacency matrices that could then be used to construct unweighted graphs. Watts et al. [1] matched some networks of interest with their random and regular equivalents. In their case, the matching procedure ensured that both random and regular networks possessed the same total number of nodes and edges as the original graph. Current practice in MRI-based neuroscience and other biomedical applications, however, tends to produce *weighted* connectivity networks. This is because MRI data take values on a continuous scale, which lends itself to the application of real-valued measures of association, such as the correlation coefficient or the synchronization likelihood among others. While different populations of unweighted networks can readily be compared by matching each network with a random network possessing an identical number of edges; there is, as yet, no consensus on how to compare populations of weighted networks in a systematic manner.

**Figure 1:**
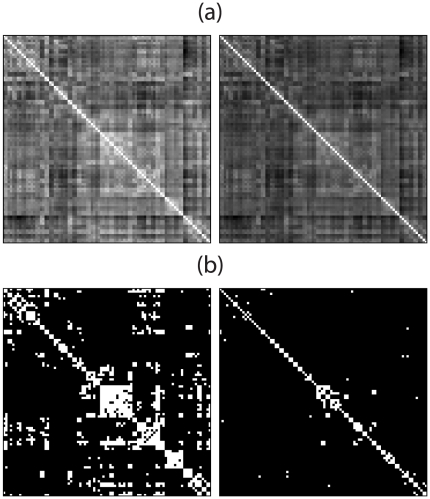
How can one disentangle differences in connectivity strength from differences in topology? In panel (a), two correlation matrices for two weighted networks differ in their average correlation strengths. In panel (b), the same correlation matrices have been thresholded at the same value, producing graphs with different cost levels. In all matrices, black indicates null values, and white denotes entries equal to unity.

This problem can be illustrated with a straightforward example. In panel (a) of Figure 1, a pair of weighted networks are represented by their correlation matrices. We are interested in comparing the topology of the corresponding weighted graphs. Since these networks differ in their mean correlation coefficients, a simple thresholding of these matrices will produce graphs of different wiring costs, i.e. different number of edges. Naturally, this thresholding is only one of the possible thresholding approaches that could be adopted. This non-uniqueness is due to the fact that graph topology is expressed in the language of discrete mathematics, whereas correlation coefficients are real-valued functions. That is, one cannot directly adopt concepts originally developed for unweighted graphs for the analysis of weighted graphs.

In this paper, we consider two main approaches to the problem of weighted network comparison. Firstly, following other authors, we evaluate the use of weighted topological metrics, which are weighted equivalents of graph-theoretical metrics for unweighted networks [19], [20]. Secondly, we consider the utilization of cost-integrated measures of topology, where all the possible wiring costs of a network are taken into account. When unweighted, a graph's wiring cost is defined as its number of edges. Integrating over wiring cost can here be interpreted in statistical terms, as an analog to the Bayesian integration of nuisance parameters. Doing so, we are averaging out the ‘uncertainty’ in the choice of a particular level of cost. Cost-integrated topological measures have been popular in the neuroscientific literature [6], [21]. However, different authors have chosen different integration intervals. We therefore explore the consequences of integrating a topological metric with respect to different subsets of the cost interval.

Note that our approach substantially differs from the one adopted by Wijk et al. [22], who proposed several formulas relating cost levels and topological measures, such as the characteristic path length or the clustering coefficient. Instead, in this paper, we are concerned with formally deriving what is the effect of integrating a particular topological measures over cost levels, in order to assess whether this is a successful manner of disentangling differences in cost from differences in topology. In particular, although Wijk et al. [22] reviews several ways of controlling for differences in cost, they do not consider cost-integration, per se. This paper can therefore be seen as a contribution to the literature on weighted network analysis, where we formally clarify the utilization of cost-integration when comparing topological metrics.

The concept of topology in the context of this paper will be defined in a quantitative manner. This should be contrasted with the qualitative definition adopted by previous authors. Wijk et al. [22], for instance, assume that networks that represent different realizations of the same ‘generative model’ should be regarded as topologically identical. Indeed, several realizations of an Erdös-Rényi model with fixed edge probability share a common generative model and therefore can be said to have an identical topology. In practice, however, such a generative model is unknown. Thus, we will refer to this type of classification as a topological taxon. A taxonony of commonly encountered networks may include the random topology of the Erdös-Rényi model, the regular lattice and the small-world topology among others. Such a nomenclature is qualitative because it relies on discrete categories. By contrast, we wish to adopt a quantitative perspective on this problem, whereby topology is operationalized in terms of specific topological properties such as the clustering coefficient (CC), for instance. In this perspective, two Erdös-Rényi models with identical edge probability may display different levels of global and local efficiencies and will therefore be considered to have distinct topological properties. Therefore, we distinguish between a qualitative approach based on topological taxonony and a quantitative approach based on topological properties. Given that generative models are latent, our quantitative definition of topology appears better suited to the empirical study and comparison of complex networks.

The above definition of network topology, however, assumes that the networks under comparison have identical numbers of vertices and edges. When this is not the case, or when one is comparing two populations of weighted networks, the question of whether or not these networks have similar topological properties becomes arduous. Our main aim, in this paper, is therefore to identify the situations within which one can safely conclude that different weighted networks share the same topological properties. In particular, we explore whether cost-integration answers this problem. Specifically, we consider whether cost-integration is a useful way of disentangling weighted cost from topology.

The paper is organized as follows. We first introduce some of the notation and basic concepts that will be used throughout the paper. We then describe the two general families of topological measures for weighted networks, which are the (i) weighted and (ii) cost-integrated metrics. For the latter, we consider different types of distribution over the cost levels. The main contribution of this paper is then reported, where different approaches to weighted network comparison are outlined, using theoretical results and simple examples. An application of these techniques to a repeated measures fMRI task investigating working memory is also described, which allows us to illustrate a Monte Carlo (MC) sampling scheme to approximate the different measures of interest. Finally, we discuss the findings of this paper in light of the current utilization of networks in the biomedical sciences. Finally, we close with a set of recommendations on how to conduct weighted network analysis in practice and how to report the findings arising from this type of research. An R package entitled NetworkAnalysis (http://CRAN.R-project.org/package=NetworkAnalysis) has been developed that makes available the methods discussed in this paper.

## Results

### Network Types and Topologies

#### Unweighted, Weighted and Fully Weighted Networks

For clarity of exposition and consistency with the previous literature, we will here employ the notation used by Kolaczyk [23]. A comprehensive introduction to the theory of complex networks can be found in Newman [24]. In the following, the terms metrics and measures will be used interchangeably to refer to a function quantifying the topological structure of a network. Our use of the terms metric and measure is unrelated to the mathematical definitions of these concepts in topology and measure theory, respectively. Similarly, we here utilize the graph-theoretical definition of the term *cost*, which is not related to its use in a probabilistic setting.

An unweighted undirected graph or network 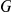 is formally defined as an ordered pair 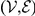, where 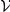 is a set of vertices, points or nodes, and 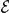 is a set of edges or connections linking pairs of nodes. Therefore 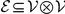, where 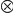 is the Cartesian product. The cardinality –i.e. the number of elements– of 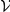 and 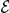 will be referred to as 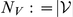 and 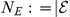, respectively, where 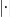 denotes the number of elements in a set, and 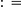 that the left-hand-side is defined as the right-hand-side. Moreover, the terms network and graph will be used interchangeably. A graph with the maximal number of edges is referred to as a complete or saturated graph. For a given network 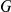, we denote the corresponding saturated graph as 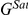. The cardinality of the edge set of 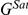 is denoted by 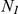 to distinguish it from 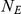. Here, the set 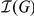, for any graph 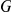 is the set of indices of all possible edges in 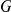. That is,
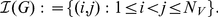
(1)This notation for the set of indices of all possible edges in 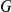 will be useful when describing the topology of 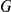 based on its shortest paths.

Weighted undirected graphs will be denoted by the triple 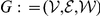, where 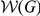 is a set of weights, whose elements are indexed by the entries in 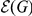, such that
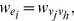
(2)for some edge 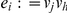. Thus, every weighted undirected graph will necessarily satisfy
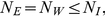
(3)where 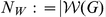. The weight set populates a symmetric matrix 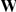, whose diagonal elements are null. Graphs that satisfy 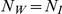 will be referred to as *fully weighted graphs*. Note that, in general, we will not draw an explicit difference between a weighted and an unweighted network through our notation. However, which one we are referring to should be understandable from the context.

There are a wide range of different weighted measures of internodal association. Our methodological development, in this paper, applies to any choice of association metric. This includes correlation coefficients, partial correlations, synchronization likelihoods and others. For simplicity, we will assume that the association weights, 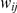
's, lie in the unit interval, 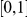. Roughly, these standardized weights, 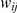, can be interpreted as the strength of the association between nodes 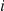 and 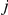, with larger values indicating a greater level of association. Such standardization can be obtained straightforwardly, in practice. For the case of the Pearson's correlation coefficient 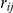, for example, the standardized weights can be defined as,
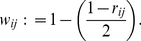
(4)Note that the use of standardized correlation coefficients suffers from two potential pitfalls. Firstly, since negative correlations are transformed into positive measures of association, it follows that we are amalgamating different subsets of edges, which may play very different roles. That is, while subnetworks of negatively correlated vertices may reflect inhibitory processes, subnetworks of positively correlated vertices may reflect excitatory processes. Secondly, since pairs of vertices linked by a small amount of correlation, either positive or negative, will be transformed to take a value close to 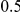; it follows that we may be introducing a spurious amount of random noise in such a weighted network analysis, as correlation coefficients close to zero are likely to be non-significant. Our approach to weighted network analysis in this paper, however, centres on thresholding the weighted networks of interest and therefore does not explicitly take into account the direction of the association. Moreover, our focus will be on fully weighted networks, such as a standardized correlation matrix, where all entries are greater than 0. Therefore, in the sequel, 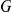 will refer to a fully weighted graph, except when specified otherwise. We will discuss the use and limitations of cost-integration for non-fully weighted graphs in the discussion.

#### Classical Measures of Network Topology

A wide range of network topological metrics have been proposed in the literature [20]. Two types of measures are generally of interest, which are sometimes referred to as (i) integration metrics and (ii) specialization metrics. The former category of topological measures quantifies a network's capacity to transfer information globally, whereas the latter reflects a network's capacity to transfer information locally. This distinction originated with the work of Watts et al. [1], who considered the characteristic path length (CPL), on one hand, and the clustering coefficient (CC), on the other hand, as measures of global and local information transfer, respectively. Although these metrics have been successfully used in a wide range of settings, Latora et al. [19] have introduced two analog metrics: the global and local efficiencies, which will be more useful in our context. These two measures retain the interpretation of the CPL and CC, while being applicable to a wider range of networks. Specifically, the global efficiency metric can be computed for any network, irrespective of its level of sparsity, which is not true for CPL. That is, the CPL becomes infinite when a graph is disconnected [25]. By contrast, the global efficiency is well-defined for any networks. For consistency, we will therefore use the global and local efficiencies to characterize global and local transfer of information in brain networks, respectively. Note, however, that analogously to the CC, the local efficiency is undefined for networks that contain isolated nodes. Thus, we set the efficiency of an isolated node to zero, which allows to integrate local efficiency over the full range of costs.

One of the remarkable aspects of global and local efficiencies is that they can both be subsumed under the general concept of information transfer efficiency, which is defined for any unweighted graph 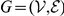 –connected or disconnected– as [19],
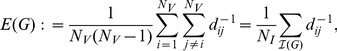
(5)where the summation over the set 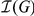 is over all the pairs of indices 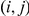 as in equation (1), and 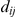 denotes the length of the shortest path between vertices 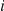 and 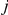 in the adjacency matrix of 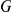, with 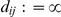 when these two nodes are not connected. The summation over 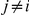 includes all indices between 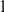 and 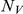 different from 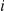. The global and local efficiencies of network 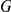 are then readily derived from equation (5), such that
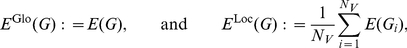
(6)where 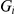 is the subgraph of 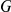 that includes all the neighbors of the 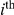 node. That is, 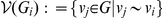, where 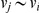 signifies that nodes 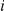 and 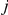 are connected. By convention, we have 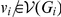 [19], [26]. Note that both global and local efficiencies are normalized quantities with values in the unit interval –that is 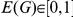. The global efficiency of a graph 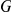 can be interpreted as the average ‘speed’ of information transfer between any pair of nodes in 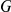, with a high value of 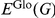 indicating a high average ‘speed’, and therefore efficient information transfer. Similarly, the local efficiency of a graph 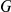 can be interpreted as the average global efficiency of the 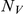 subgraphs of 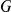, where again a high value for 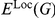 implies efficient local information transfer, on average.

We have used 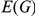 to denote the efficiency metric of the unweighted graph 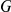. This should be distinguished from the graph-theoretical concept of the edge set, which we have denoted 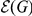. Since both quantities are functions of 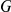, we have emphasized this distinction through our notation. Note also that we will make use of the expectation operator from probability theory, which will be denoted by 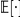. For simplicity, all our development, examples and technical results will be based on the general efficiency described in equation (5). However, these methods could readily be extended to both global and local efficiencies. In fact, most of our discussion applies to all topological metrics that can be computed for any level of sparsity. We will discuss the generalization of our results to other topological measures in the Discussion section.

#### Cost and Weighted Cost

In network analysis, it is often of interest to quantify the cost or wiring cost of an unweighted graph. In this section, we extend this concept to weighted networks. This generalized version of cost will be termed the weighted cost or weighted density.

The cost or density, 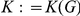, of an unweighted network 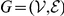 quantifies the relative number of connections in 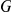 as a proportion of the number of edges contained in the 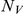-matched saturated network 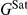. That is,
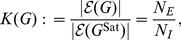
(7)where 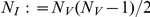. The computation of the cost of a network 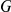 implicitly refers to the adjacency matrix 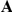 of that network. Hence, we can reformulate the definition in equation (7) by explicitly using 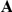 as follows,
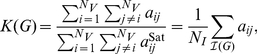
(8)where the 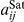
's denote the elements of the adjacency matrix 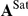, which represents a saturated network of 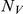 nodes.

Similarly, it will be of interest to quantify the cost of a weighted network, which will be referred to as 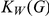. We define it by generalizing the relationship between an unweighted graph and its adjacency matrix in order to apply it to weighted graphs and their association matrices. However, to extend the concept of cost to a real-valued association matrix, say 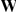, we need to formalize what we mean by a *saturated weighted graph*. A natural choice is to define 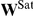 as a matrix of order 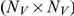 with unit entries. Formally, 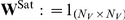. Using this saturated association matrix, we can now define the cost of a weighted graph as follows,
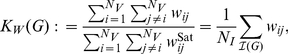
(9)where 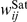
's are the elements of 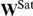. The non-standardized version of the cost of a weighted network in equation (9) was introduced by Fallani et al. [11]. Thus, the weighted cost of 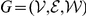 is the mean of the off-diagonal elements in 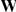, populated by the set 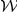. This is reminiscent of our starting point in equation (7), where the same observation can be made about unweighted networks. In the sequel, the concept of weighted cost will be used interchangeably with the phrase *connectivity strength*. Note that depending upon which standardization one chooses, one may obtain different types of weighted costs. In particular, 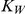 could also be standardized with respect to the number of elements in 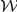. This would produce a different measure. In this paper, we will assume that the networks under consideration are fully weighted, such that 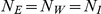, and therefore these two types of weighted costs are equivalent.

There is currently no guidance in the literature on how to quantify the topological aspects of a weighted network. We review here two approaches to this problem in the following two sections: (i) weighted, and (ii) cost-integrated metrics of network topology. We describe and define these two families of measures, in turn.

### Weighted Measures

A natural approach to the problem of quantifying the topology of weighted networks is to translate unweighted measures, such as efficiency metrics, for example, into a weighted format. This is a very general procedure, which has been introduced by several authors including Latora et al. [19] and Rubinov et al. [20]. Weighted versions of classical metrics commonly rely on the definition of a weighted shortest path. For unweighted networks, the shortest path 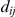 between nodes 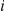 and 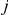 in 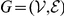 is defined as the following minimization,
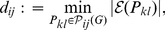
(10)where 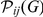 is the set of all paths between nodes 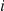 and 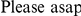 that are subgraphs of 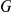. A subgraph 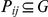 is a path if and only if 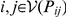 such that
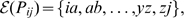
(11)where each pair of letters stands for an edge. One can similarly define a *weighted shortest path*, 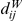, for some weighted graph 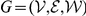 as follows,
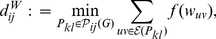
(12)where the weighted edge set of a path now takes the form,
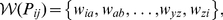
(13)using the notational convention introduced in equation (2). Since we have normalized the association weights, 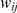
's, the real-valued function 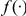 is restricted to a map of the form 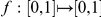. A convenient choice of 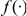 is the inverse function, 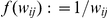. It now suffices to use our chosen definition of the weighted shortest path 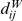, in order to obtain a weighted version of the general efficiency metric in equation (5), which gives
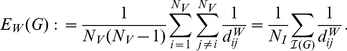
(14)Note that weighted efficiency is here bounded between 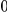 and 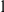. Since the standardized association weights take values in 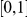, it then follows that 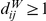, and therefore 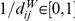, for every pair of vertices.

### Cost-integrated Measures

A second approach to the problem of quantifying the topology of weighted networks proceeds by integrating the metric of interest with respect to cost. Here, some authors have integrated over a subset of the cost range [7], whereas others have integrated over the entire cost domain [27]. This second family of metrics will be referred to as cost-integrated measures. Given a weighted graph 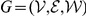, the cost-integrated version of a topological metric 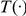 is defined as follows,
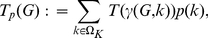
(15)where cost is treated as a discrete random variable 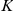, with realizations in lower case, and 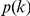 denotes the probability mass function of 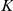. Since 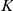 is discrete, it can only take a countably finite number of values, which is the following set,
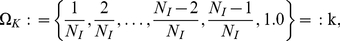
(16)where, as before, 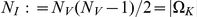. It will be useful to treat 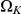 as an ordered set, 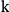, whose elements, 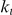
's, are arranged in increasing order and indexed by 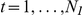. The function 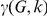 in equation (15) is a thresholding function, which takes a weighted undirected network and a level of wiring cost as arguments, and returns an *unweighted* network. We defer a full discussion of 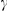 to Methods B, where we describe its definition in more detail. This function is based on the percentile ranks of the elements of 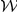, where tied ranks are resolved by assigning the corresponding ordering of the elements' indices. Since 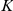 is treated as a discrete random variable, we can define its probability mass function. We will here consider two different choices for 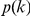: (i) a uniform distribution on 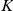 and (ii) the use of a Beta-binomial distribution on 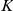.

#### Uniform Distribution on 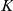


Firstly, as there is no prior knowledge about which values of 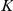 should be favored, one may choose to specify a uniform distribution over 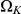. In equation (16), we have excluded the null cost for standardization purposes. Since any edge-based topology of interest will be zero when 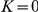, this particular value is irrelevant when comparing different populations of networks. In example 3, we will also see that this exclusion of the point mass at 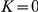 ensures a more satisfying standardization of 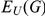. As no particular cost levels are favored, 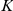 is given a discrete uniform distribution, such that
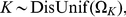
(17)where each element of 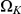 has an identical probability of occurrence, which, in our case, is equivalent to
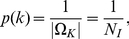
(18)for every 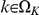. The theoretical integration in equation (15) is therefore a weighted summation over a finite set of atoms [28], and may be computed as follows. The cost-integrated version of the general efficiency in equation (5) then becomes:
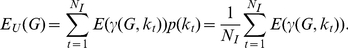
(19)where the index 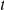 runs over the elements of 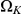 described in (16).

#### Beta-binomial Distribution on 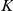


**Figure 2:**
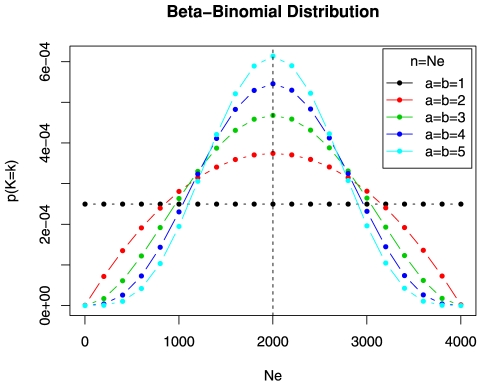
Symmetric versions of the Beta-binomial distribution for different choices of parameters, with 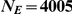.

Secondly, one may also choose to favor different portions of the domain of 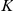. This can be formally conducted by specifying a Beta-binomial distribution on 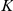. The Beta-binomial distribution is particularly suited to this task because it can be regarded as a discrete version of the Beta distribution over a discrete interval, and can be parametrized in order to de-emphasize the importance of the topologies situated at the ends of the cost domain: i.e. for very low and very high costs. This is a distribution, which commonly arises in the context of Bayesian statistics, as the marginal likelihood of a hierarchical model with binomial likelihood and Beta prior [29]. Since realizations from a Beta-binomial distribution represent the number of successes on a sequence of Bernoulli trials, it follows that we here need to consider the probability of the number of edges, 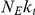. Thus, we have the following definition linking the probability mass function of 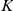 with the one of the number of edges,
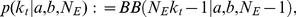
(20)for every 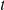 and where the Beta-binomial distribution is given the following parametrization,
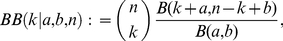
(21)with 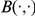 denoting the Beta function. In equation (20), we have subtracted 1 from all the realizations of the Beta-binomial distribution in order to restrict the domain of that distribution to the number of elements in 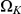, as we have excluded the null cost. Thus, this distribution weights each 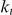 according to the distribution controlled by 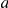 and 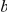. The corresponding formulae for the general efficiency is therefore,
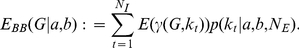
(22)In figure 2, different distributions of 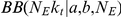 have been plotted for different choices of 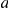 and 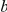, while 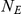 has been chosen to reflect the size of the functional brain networks. The values given to the parameters 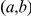 determine which cost levels are upweighted. In particular, one can observe that 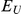 can be recovered as a special case of 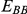 by selecting 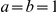, as shown in figure 2. We have here restricted ourselves to symmetric versions of the Beta-binomial distributions, but asymmetric choices are also possible.

#### Cost-integration over a Subset of the Costs

More generally, cost-integrated metrics can be defined with respect to a subset of the cost regimen. We here illustrate this approach for the case of uniform cost-integration. This perspective on the problem of weighted network comparison has been utilized by several authors [5], [7], [30]. In our notation, a subset of the cost levels will be indicated by an interval of the form 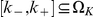, which refers to a finite number of values of 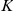, satisfying 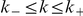. Integration over that subset is then defined as
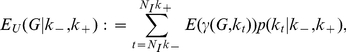
(23)where the probability mass function on 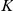 is normalized with respect to the chosen domain of integration 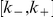, such that 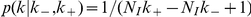, for every 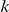 in that interval. The computational formula for this generalization of equation (19) is then given by
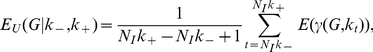
(24)which follows from 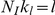, using the definition of cost in equation (7). Note that the value of the conditional probability 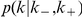 will be different if semi-open intervals such as 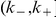 are considered, instead of closed ones. This is due to the fact that the interval of interest is over a set of discrete values, as opposed to a subset of the real line. As a special case, this notation can also handle the estimation of a particular topological metric at a single cost level, say 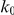. In such cases, the interval of interest becomes 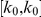. Our notation makes explicit the fact that integration over a subset of the full cost regimen, is conditional on the choice of such a subset.

Since 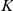 has been treated as a random variable and because 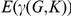 is a function of 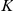, it follows that 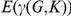 is also a random variable. The integral 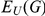 can therefore be seen as the expectation of 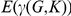 with respect to the distribution of 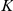. This probabilistic treatment of cost-integrated metrics will be particularly helpful when considering how to estimate these quantities, as a Monte Carlo (MC) sampling scheme can readily be devised in order to approximate 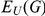, when the network of interest is too large to be computed exactly. More details about this sampling scheme are given in Methods A.

### Pros and Cons of Integrating over Cost Levels

We now turn to the main question tackled in this paper: Is it useful to integrate over the different cost levels of a particular weighted network? In order to answer this question, we briefly consider some of the alternatives to this approach. This consists of (i) fixing a cutoff point, (ii) fixing a cost regimen, (iii) integrating over all cost levels, and (iv) directly using weighted topological metrics. Our comparison of these four approaches is substantiated by some simple examples, synthetic data sets, and theoretical results. For convenience, we will solely treat the case of two weighted networks in this section. Extensions of these ideas to the case of several populations of networks are discussed in the Discussion.

#### Fixing a Cutoff Threshold

The simplest way of comparing the topology of weighted networks is to threshold the corresponding association matrices at a specific value, and evaluate the resulting discrete topologies. It is instructive to study the consequences of such a naive thresholding on two networks with proportional association matrices, as we describe in the following example.

**Example 1** Let two weighted networks 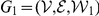 and 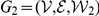, with standardized association matrices denoted 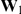 and 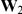, respectively; such that every 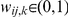 where 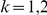 labels the two graphs under scrutiny. In addition, assume that
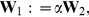
(25)where 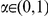 is a scalar. That is, the association matrix of 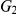 is simply proportional to that of 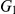. Two such association matrices have been discussed in the introduction and were illustrated in panel (a) of Figure 1. Note that the relationship in equation (25) implies that the diagonal elements of 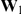 are not standardized to 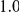. However, the topology and cost of weighted networks solely depend on the off-diagonal elements of such association matrices. Therefore, differences in the diagonal elements do not pertain to this discussion. Interestingly, it is easy to show that proportionality in association matrices implies proportionality in weighted cost. Using equation (9), we have
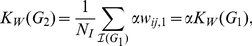
(26)since 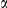 is applied elementwise. Therefore, 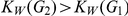 as by assumption 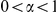.

A naive approach to the problem of comparing the topologies of these two networks may proceed by thresholding 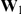 and 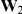 at a particular value, say 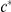, as was done in the introduction. If we compare these networks in terms of global efficiency, straightforward computation of the two corresponding quantities shows that we necessarily have
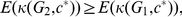
(27)for any 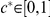, where 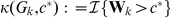. This follows since 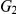, thresholded at 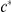 has all the edges of 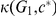, as well as additional links owing to its weighted cost being higher. The relationship in equation (27) is then deduced from the monotonicity of the efficiency function with respect to cost. Note that these inequalities would hold for both local and global efficiencies, or any other topological metric, which is a monotonic increasing function of the cost level. Therefore, example 1 has shown that thresholding weighted graphs at a fixed cutoff point is misleading, since graphs with higher weighted cost will tend to be classified as having higher levels of global efficiency. This problem can be remedied by fixing cost levels instead of cutoff points.

#### Fixing a Cost Level

A natural approach to the problem of separating cost from topology is to choose a particular cost level. This may be a single value or a subset of the cost regimen. Such a strategy has been adopted by several authors [5], [7], [30]. One of the original justifications for conditioning over a subset of the cost regimen was that topological metrics such as CPL or CC cannot be computed for disconnected networks, thereby making it impossible to calculate these quantities for small cost levels. However, since comparable global and local topological properties can also be measured using the efficiency metrics introduced by Latora et al. [19], such problems do not arise when using these topological metrics. We illustrate the consequences of integrating over a subset of the range of 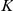 with a real data example, where the original data has been transformed. We have constructed a pathological case, which shows that integrating over a subset of the cost levels can fail to distinguish between topologically distinct weighted networks.

**Figure 3:**
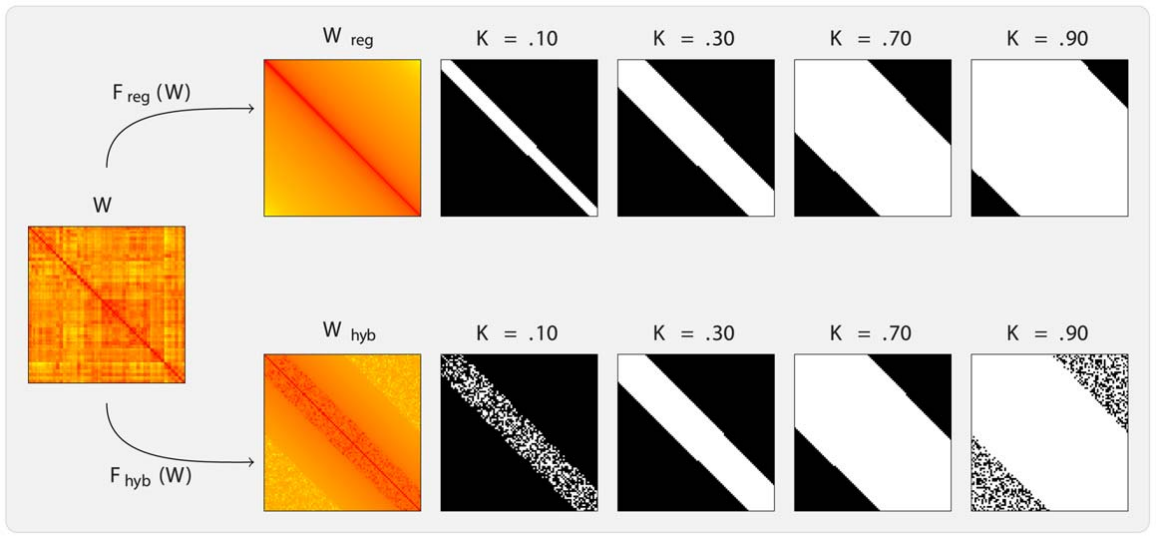
Simulation framework for example 2. The small-world correlation matrix 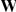 is transformed into a regular and a hybrid matrix, denoted 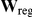 and 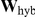, respectively. The regular matrix exhibits a lattice-like topology throughout its cost range, whereas 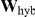 consists of alternating topological layers of random and regular structures. The entries in both matrices have been arranged in decreasing order from the diagonal, to facilitate visualization.

**Example 2** We here consider a single functional connectivity matrix 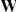, corresponding to the mean statistical parametric network (SPN) of a previously published data set [31]. The matrix 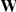 was transformed in order to produce two other matrices with either a regular or a hybrid structure, denoted by 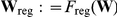 and 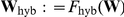, respectively. The functions 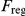 and 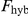 simply re-organize the position of the entries in 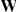, as can be seen from Figure 3. The choice of these transformations was constrained by the following prescriptions,
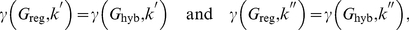
(28)for cost levels 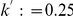 and 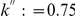, respectively. That is, the adjacency matrices corresponding to costs 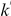 and 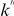 are identical for 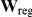 and 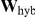. The effect of the functions 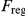 and 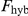 was to create different layers of topological structures that vary according to wiring cost. The hybrid matrix was composed of alternating layers of random and regular topologies. Roughly, the three layers of the hybrid network corresponding to an hybrid association matrix can approximately be described as follows,
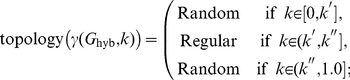
(29)for every 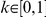, where 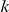 can only take a finite number of values in the unit interval. The regular matrix, by contrast, was built as three layers of regular topologies. That is,
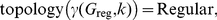
(30)for every 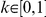. The random and regular layers were constructed in a standard fashion [31]. Matrices 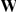, 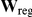 and 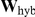 corresponding to weight sets 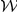, 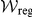 and 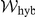, are represented in Figure 3 with the corresponding adjacency matrices resulting from different choices of cost levels.

By construction, the weighted graphs 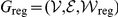 and 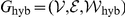 have identical levels of general efficiency for the cost levels comprised in the interval 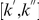. Therefore, integrating over that interval gives the same result for both graphs:
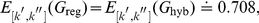
(31)where 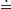 means approximately. By contrast, the general efficiencies of these two networks differ substantially when uniformly integrating over the full range of cost, i.e. 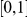. This gives
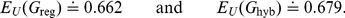
(32)This is as expected, since the hybrid network has several layers of random topologies, which renders it more globally efficient than 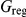.

Example 2 illustrates the problems associated with integrating over a subset of the cost regimen. By doing so, we are potentially omitting substantial topological differences between the networks of interest at other cost levels. The difference in 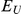 between 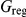 and 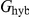 reported in that counterexample may not appear very large. However, these two networks could have represented the mean networks of two populations of interest. Providing that the pool of subjects is sufficiently large, such topological differences could be found to be statistically significant. By contrast, comparison of these two networks on the basis of the full cost regimen yielded answers, which were exactly identical, thus nullifying any statistical test of group differences. Naturally, this example could have been constructed in the opposite direction in order to show that networks that seem to differ topologically for some cost subsets are, in fact, identical when uniformly integrating over the full cost regimen.

Fixing a cost level or a subset of the cost regimen therefore suffers from two main problems. Firstly, the arbitrariness of the choice of a specific cost subset will generally be difficult to justify from either a theoretical or a practical perspective. Secondly, as we have illustrated with example 2, considering only a subset of the cost potentially omits topological differences, which are solely visible at other cost levels. Thus, any network analysis using this strategy can only draw conclusions that are *conditional* on the choice of cost subset, and this dependence should be made explicit when reporting the results of such analyses. Nonetheless, fixing a particular cost subset successfully satisfies one of our desiderata, which was to disentangle differences in cost from differences in topology. That is, weighted networks' topologies can be compared irrespective of cost differences, by conditioning on some subset of the cost levels. This invariance property will be made mathematically more precise in the next section.

#### Integrating over Cost levels

From a statistical perspective, the problem of isolating topology from connectivity strength may be reformulated as evaluating topological differences while ‘controlling’ for cost, where these two quantities are treated as random variables. A natural starting point is to consider weighted networks whose association matrices are proportional to each other, as in the ensuing example.

**Example 3** As a simple example, consider the following problem. Let two weighted networks 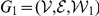 and 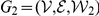, be characterized by the following standardized association matrices:
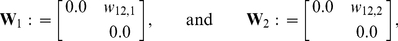
(33)where we assume that 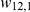 and 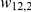 are comprised in the open interval 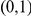. Here, there are only two levels of cost, 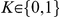. Trivially, 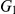 and 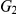 can therefore be shown to exhibit identical general efficiency for these two cost levels. Since our proposed formula for cost-integrated topological measures in equation (19) does not include the null cost, we simply have 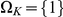, which implies that both graphs attain the maximal level of uniformly cost-integrated efficiency. That is, considering a uniform distribution over 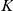, we have
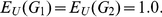
(34)This simple example serves as a justification for our exclusion of the null cost from the set 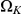 in equation (16). Including the null cost would result in 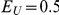 for these two basic networks, which does not appear satisfying. Crucially, the equality in (34) does not depend on the relationship between 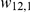 and 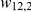. That is, differences in weighted cost have no impact on cost-integrated topology. We now return to the case studied in example 1 in order to elucidate the exact effect of cost-integration.

**Example 1** (Continued) In this example, we considered two networks with proportional association matrices, satisfying 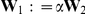. An application of the uniformly cost-integrated metrics described in equation (19) to the networks of this example gives the following equalities,
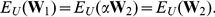
(35)That is, when uniformly integrating with respect to the cost levels, we are evaluating the efficiencies of 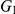 and 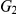 at 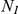 discrete points. At each of these points, the efficiency of the two networks will be identical, because 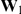 is proportional to 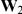 and therefore the same sets of edges will be selected. Thus, 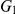 and 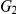 have identical cost-integrated efficiencies.

The equalities derived in these two examples can be shown to hold in a more general sense. The invariance of cost-integrated efficiency turns out to be true for any monotonic (increasing or decreasing) transformation of the association matrix and applies to any topological metric, 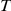, that takes an unweighted graph as an argument, as formally stated in the following result.

**Proposition 1** *Let a weighted undirected graph* 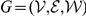 *. For any monotonic function* 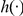 *acting elementwise on a real-valued matrix,* 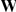 *, corresponding to the weight set* 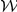 *, and any topological metric* 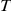 *, the cost-integrated version of that metric, denoted* 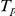 *, satisfies*

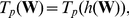
(36)where we have used the association matrix, 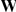, as a proxy notation for graph 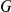.

A proof of this result is provided in Methods B. It relies on the idea that the evaluation of a weighted network solely depends on the ranking of the off-diagonal elements of 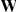 (i.e. the ranking of the elements in 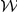), and that the ranks of a set of values are independent of a monotonic transformation of these values. Note that the arguments used in Methods B do not rely on the definition of 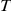, nor on the choice of 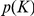. Therefore, proposition 1 is true for any cost-integrated topological metrics –i.e. a metric originally defined in a discrete setting for an unweighted graph, and integrated with respect to cost, when applied to a weighted network. Note also that proposition 1 only holds for all levels of sparsity of 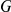 if the thresholding function 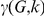 used in the computation of a cost-integrated metric preserves the original ordering of elements in 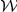 with tied ranks, using their indices. In general, however, sparse networks may better be dealt with, in this context, by adjusting the size of the integration domain.

Proposition 1 encapsulates both the advantages and limitations of cost-integrated topological metrics. Two weighted networks, whose topologies are roughly identical at every cost level will be given identical scores under this family of metrics, irrespective of cost differences. Cost-integrated metrics are therefore successful at winnowing topology from connectivity strength. Another singular advantage of this approach is that we obtain a measure, which is invariant under any normalization or standardization of the original data. That is, any functions that simply rescale or shift the association weights, in order to ensure that they are comprised in the unit interval, for instance, will have no effect on the value of the cost-integrated topological measures.

However, proposition 1 also demonstrates the limitation of such an approach. One can easily see that such cost-integration will potentially mask some cost-specific topological differences, as illustrated in example 2. In addition, when cost-integrated topological metrics are used for network comparison, this requires that the sizes of the weight sets of different networks are identical. Similarly, the presence of multiplicities in the ranks of the weights may also cause problems, as this would artificially induce a random topological structure, since weights with equal ranks would be randomly allocated to different cost levels. We will further discuss these limitations in the conclusion of this paper.

#### Using a Weighted Metric

A seemingly natural way of amalgamating connectivity strength and topological characteristics is by directly considering weighted topological metrics, such as the weighted global efficiency, 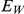, introduced in equation (14). Unfortunately, we here prove that such an approach suffers from a serious limitation, which could potentially dissuade researchers from using this particular type of metrics. With the next proposition, we show that in a wide range of settings, the weighted efficiency is simply equivalent to the weighted cost of the graph of interest.

**Proposition 2** *For any weighted graph* 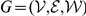 *, whose weight set is denoted by* 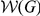 *, if we have*

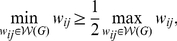
(37)then
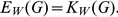
(38)


This result can be proved by contradiction, as demonstrated in Methods C. The hypothesis in proposition 2 may at first appear relatively stringent. However, it will encompass a wide range of experimental situations. For the real data set described in example 2, the difference between 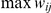 and 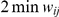 is close to, but not exactly, zero. However, we nonetheless have 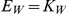, for that example. Thus, the added value of using the weighted efficiency will, in general, be questionable since there exists a strong relationship between this topological measure and a simple average of the edge weights.

These theoretical results and associated counterexamples have therefore highlighted the limitations of various approaches to the problem of disentangling differences in cost from differences in topology. As a result, when comparing several populations of networks, we recommend the reporting of differences in weighted costs and differences in cost-integrated topological measures. We illustrate this approach with a re-analysis of a previously published fMRI data set.

### 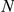-back Working Memory Data Set

In this section, we illustrate our theoretical results with a previously analyzed data set of a working memory task based on functional Magnetic Resonance Imaging (fMRI) data [31]. In particular, we use this data set for testing our proposed MC sampling procedure and for comparing a graph's weighted cost with its cost-integrated and weighted global efficiencies.

#### Description

Ginestet et al. [31] considered topological changes in functional brain networks under different levels of cognitive load. Here, we give a cursory description of the experimental procedure used in this study and refer the reader to the original paper for the full technical details. Ginestet et al. [31] constructed networks on the basis of fMRI data gathered from 43 healthy adults undergoing a working memory task known as the 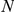-back paradigm. Echo planar imaging data quality was assessed using an automated technique [32]. In this experiment, subjects were shown one letter every two seconds, and were asked to monitor the stimuli, in order to indicate by the push of a button whether the current letter was identical to the one presented 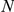 trials previously, where 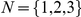. A control or null condition was also included, the 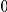-back task, which consisted of simply indicating whether the current letter was an X. In this experiment, the subject-specific fMRI images were parcellated into 90 regions of interest using the Anatomical Automatic Labelling (AAL) template [33]. The BOLD time series were averaged for each AAL region. These regional mean time series were then wavelet decomposed. Wavelet coefficients in the low frequency range (0.01–0.03 Hz) were selected for the main network analysis [6]. Since the 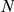-back paradigm contains four experimental levels, we decomposed these time series into blocks corresponding to each 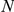-back condition. As each condition was repeated more than once, these blocks were then concatenated. Note that this sequence of processing steps involving wavelet decomposition immediately followed by block concatenation was studied by Ginestet et al. [31] using simulated data, and was not found to bias the results of the final network analysis.

**Figure 4:**
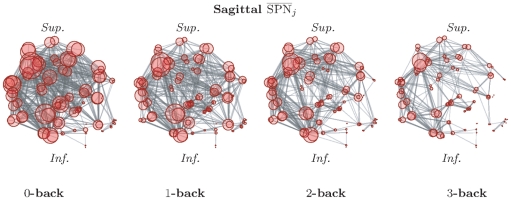
Mean Statistical Parametric Networks (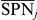) over the 4 levels of the 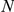-back task, in the sagittal plane, based on wavelet coefficients in the 0.01–0.03 Hz frequency band, with FDR correction (base rate 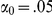). Locations of the nodes correspond to the stereotaxic centroids of the corresponding cortical regions. The inferior–superior orientation axis is indicated in italics. The size of each node is proportional to its degree.

Vertices in these subject-specific functional networks were chosen to be the 90 AAL regions, and the edges were constructed by computing pairwise correlations between each condition-specific time series of wavelet coefficients. The results of this construction can be summarized using Statistical Parametric Networks (SPNs), as illustrated in Figure 4 [31]. SPNs are estimated using a mass-univariate approach, where the edges in a population of subject-specific networks are tested for significance using a mixed-effects model, and then thresholded using the false discovery rate [34], [35]. SPNs can be constructed using functions made freely available through the R package NetworkAnalysis (http://CRAN.R-project.org/package=NetworkAnalysis). From Figure 4, one can observe that the connectivity strength (i.e. weighted cost or averaged correlation coefficient) of the functional networks in each condition tend to diminish as subjects experience greater cognitive load.

#### Monte Carlo (MC) Estimation

**Figure 5:**
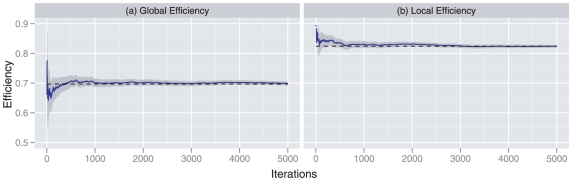
Running means of Monte Carlo (MC) estimates for uniformly cost-integrated global and local efficiencies in panels (a) and (b), respectively, for the 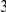-back network described in example 2. The grey ribbon represents the variability of these estimators at each 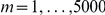, using twice the MC standard error. That is, 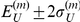 for both global and local efficiencies. The dashed lines indicate the exact value of 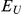. See Methods A for details.

A full description of the theory supporting MC estimation in this context is provided in Methods A. MC techniques are here used to speed up the computation required when estimating our proposed cost-integrated measures. Figure 5 shows the convergence of 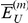 to 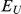, for a medium-sized weighted network derived from fMRI data on the working memory task described in example 2. The results are provided for both global and local efficiencies. Each plot in Figure 5 shows the running mean plus or minus twice the running MC standard error, which are defined for the uniformly cost-integrated global efficiency, as 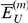 and 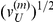, respectively, where 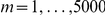. (See Methods A for details.) In Figure 5, we also report the exact values of 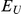 using formula (19) by dashed lines.

In all the cases studied, the MC estimates compared favorably with the exact integrals after approximately a quarter of the number of computations required for the exact calculations. That is, the exact derivation of 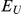 necessitates 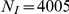 evaluations of the global or local efficiency. By contrast, MC estimates based on approximately 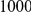 samples appear to provide reasonably good approximations of these quantities, as indicated by the small MC standard error. This constitutes a non-negligible computational gain. The MC standard error, which is derived as a by-product of these computations could then be used as an indicator of the uncertainty associated with these estimates in a Bayesian hierarchical model, where uncertainty is propagated from the data to the population's parameters of interest.

A simple alternative to MC averaging, in our context, would be to construct a mesh of the unit interval and to approximate the desired integral by a weighted sum of the values of the topological metric of interest at the midpoints of that mesh. The latter method is generally referred to as the Gauss-Kronrod quadrature formula [36]. While this method is very efficient for simple functions, it becomes rapidly unwieldy for complex ones, as it requires an increasingly refined mesh to ensure good interpolation. Moreover, since the Gauss-Kronrod is a deterministic algorithm, it does not provide a measure of the accuracy of the estimation. By contrast, a MC approach ensures asymptotic convergence for any level of complexity and also produces precise confidence bands. (See Methods A for details.)

#### Evaluation and Comparison

Following the statistical framework used in the original analysis of this data set [31], we tested for the statistical significance of the 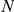-back factor on different topological metrics using a mixed-effects model. We here have 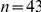 subjects and 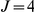 experimental conditions. Using the formalism introduced by Laird et al. [37], we have
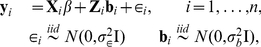
(39)where 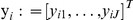 is a subject-specific vector of topological metrics of interest, 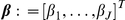 is a vector of fixed effect, which do not vary over subjects, 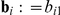 is a subject-specific random effect and 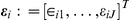 are the residuals. Finally, the matrices 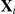
's and 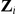
's are given the following specification,
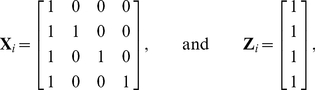
(40)for every 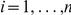. The effect of the 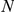-back factor was then evaluated using Wald's 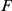-test. All these analyses were conducted within the R environment using the lme4 package [38]. Note that the model used here is slightly simpler than the one used in Ginestet et al. [31], as the present mixed-effect model was found to be better identified than the growth curve model utilized in the original analysis.

**Figure 6:**
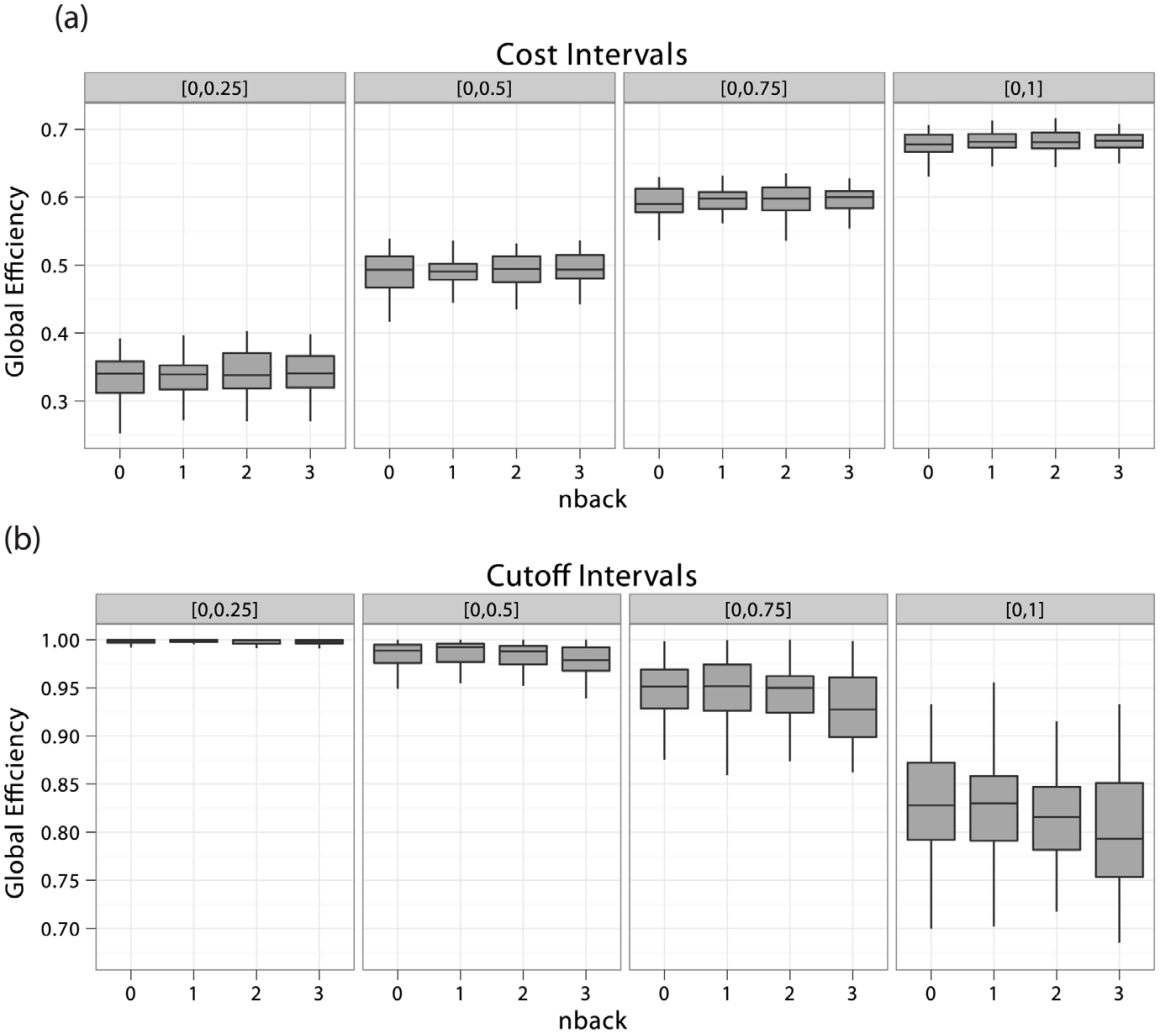
Box plots of cost-integrated global efficiencies. In panel (a), uniformly cost-integrated and in panel (b), Beta-binomial cost-integrated global efficiencies of fMRI 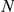-back networks for four different domains of integration and different choices of parameters are represented. These integrals were estimated using MC approximation over 1,000 samples for each of the 43 subjects in each of the four experimental conditions. Note that different domains of cost-integration do not induce any differences in the effect of the experimental factor, in panel (a). However, the use of the Beta-binomial distribution seems to indicate that a gradual increase in global efficiency follows an increase in cognitive load, in panel (b). However, these relationships did not reach statistical significance (see Table 1).

In Figure 6, we report the cost–integrated global efficiencies for this experiment. For illustrative purposes, we have computed these quantities for four different choices of domains of integration. The 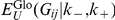 were here estimated using 1,000 MC samples for each subject in each 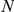-back condition. In panel (a), one can observe a clear increase of the cost-integrated global efficiencies as we increase the size of the domain of integration, due to the monotonicity of global efficiency with respect to cost. This is a standard property of global efficiency: as graphs become denser, their diameter tends to diminish [39]. In Figure 6, one can also note the dependence of the inter-subject variability of the cost–integrated metrics on the chosen domain of integration.

We therefore tested for the effect of the 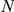-back factor on the topological metrics of interest, given different domains of integration, in order to evaluate whether such a choice of domain has a systematic impact on the effect of the experimental factor. These tests are based on the mixed-effects model described in equation (39), and we have reported the results of these statistical tests in Table 1. These results do not indicate that the choice of different domains of integration or the choice of different specifications of the Beta-binomial distribution yield systematic biases in statistical inference. As was reported by Ginestet et al. [31], the weighted cost was found to be systematically affected by the 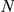-back factor 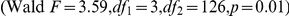. However, none of the cost-integrated global efficiencies appeared to be significantly influenced by the experimental factor. Most importantly, neither the use of different domains of integration nor the specification of different parameters for the Beta-binomial distribution seemed to affect the results. Integration over the entire cost domain, however, resulted in a larger 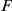-statistic, which may be explained by the lower amount of variability characterizing cost-integration over larger cost domains, as can be observed in Figure 6. In panel (b), cost-integrated efficiencies with respect to the Beta-binomial distribution for several choices of parameters indicate that a possible relationship between global efficiency and cognitive load may exist. However, this relationship did not reach statistical significance, as can be seen from Table 1. It therefore appears that although different choices of probability mass functions on 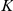 yielded slightly different results, the overall analysis seems to indicate that the experimental factor was not a predictor of global efficiency.

**Table 1:** Statistical inference for the mixed-effects model described in equation (39).

*Outcome Variable*	*Cost Domain*	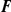 -statistic	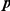 -value
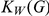			
Weighted Cost		3.59	0.01
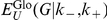			
Cost-integrated	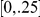	0.34	0.79
Cost-integrated	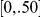	0.24	0.86
Cost-integrated	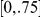	0.40	0.75
Cost-integrated	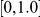	1.09	0.35
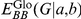			
Beta-binomial, 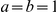	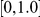	1.09	0.35
Beta-binomial, 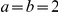	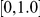	0.94	0.42
Beta-binomial, 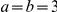	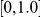	0.97	0.41
Beta-binomial, 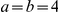	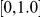	1.37	0.25

Testing of the effect of the 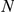-back factor on global efficiency. For uniformly cost-integrated (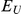) global efficiencies, we have separately tested four different domains of integration, whereas for Beta-binomial (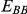) cost-integration, we have considered four different specifications of the parameters of the Beta-binomial distribution.

**Figure 7:**
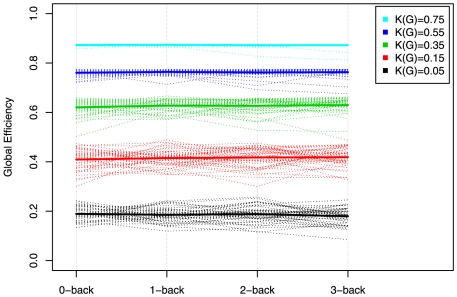
Interaction plots of cost-dependent global efficiencies of fMRI networks with respect to the levels of the 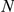-back factor. We here consider five different costs 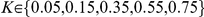. The dashed lines represents the cost-specific global efficiencies for each subject, whereas the plain line represents cost-specific global efficiencies averaged over the 43 subjects. The flatness of the lines at each cost levels suggests that the experimental factor has little effect on the topological structure of these networks.

In addition, in Table 1, we have also reported the 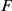-statistic for the effect of the 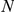-back factor on the weighted cost. The subject-specific network's weighted costs were found to be significantly influenced by the level of the experimental factor, as is immediately visible from the mean SPNs reported in Figure 4. The separation of the differences in cost from the differences in topology that results from the use of a cost-integrated topological metric is best illustrated by the interaction plots in Figure 7, where ensembles of global efficiencies corresponding to different costs are represented for the four levels of the experimental factors. Note that, here, we are reporting the efficiency metrics for a single level of cost, not integrated over a subset of the cost regimen as was done in Figure 6. This is a visual depiction of the 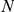-back factor that corroborates the conclusions reached using cost-integrated topological metrics, which stated that topology, as measured by global efficiency, does not significantly vary with the experimental factor.

## Discussion

This paper has investigated the effect of thresholding matrices of correlation coefficients or other measures of association for the purpose of producing simple unweighted graphs. On the basis of this analysis, and the examples studied in this paper, we make the following methodological recommendations to researchers intending to compare the topological properties of two or more populations of weighted networks.

### Summary and Recommendations

We here summarize the main findings of this paper: (i) fixing a cutoff threshold is not satisfactory, because this is fully determined by differences in connectivity strength, as we have shown previously; (ii.) fixing a subset of cost levels is not satisfactory, because this potentially omits topological differences at other cost levels; (iii) integrating over the entire cost regimen successfully disentangles connectivity strength from topology up to monotonic transformations. Specifically, such metrics are invariant to monotonic transformations of the association weights; and (iv.) the weighted topological metrics, such as 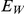, appear to be too closely related to weighted costs.

From a methodological perspective, we therefore recommend the following. As a preliminary step, it is good practice to standardize the association weights, in order to obtain 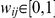 for all 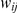
's, with large values of the weights corresponding to strong associations, although some care must be taken in the interpretation of the resulting standardized weights. This may facilitate comparison across separate network analyses, and ease the interpretation of the results. Secondly, the weighted cost, i.e. connectivity strength, of the networks of interest can then be computed for all networks. This is central to the rest of the analysis, and should be conducted systematically. Moreover, quantitative differences in connectivity strength *per se* are informative about the brain processes at hand, and their experimental relevance should not be neglected. Thirdly, population differences in cost-integrated topological metrics may then be evaluated. This will indicate whether the topologies of the populations under scrutiny vary significantly irrespective of their differences in connectivity strength. This aspect of network analysis could be regarded as qualitative, as this reflects the networks' architectural properties. In Table 1 and Figure 6, we seen have that different choices of parameters for the Beta-binomial distribution can lead to different inferential results. Therefore, it may be appropriate to report such results for different choices of distributions over 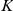, in order to assess the sensitivity of these results to such changes. We now expand and discuss some of the remarks that were made in the Results section.

### Limitations of Cost-integration

As with any form of averaging, cost-integration ignores cost-specific topological differences. Networks 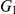 and 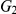, in example 1, differ in connectivity strength and these differences may also be expressed through their cost-dependent respective topologies. That is, as illustrated in example 2, certain graphs may not exhibit the same topological structure at different cost levels, and therefore integrating over cost may potentially mask these subtle topological differences. Another potential problem with cost-integrated quantities is that they may be expensive computationally. The number of possible cost levels increases at rate 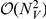 with respect to the number of vertices in the networks of interest. In Methods A, however, we show how such integrals can be estimated through MC sampling, which can substantially diminish the required computations.

Another potential pitfall which is not directly visible from proposition 1 is that the use of cost-integration for the comparison of several populations of networks requires these networks to have the same number of positive weights. That is, to be comparable two networks do not simply need to possess the same number of vertices, i.e. 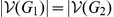, but also should have the have the same number of weights, i.e. 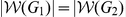. In this paper, we have re-analyzed an fMRI data set, based on correlation matrices, which produce fully weighted networks, for which 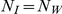 for every subjects. However, when such a condition does not hold, we recommend the selection of a domain of integration that corresponds to the smallest common denominator. That is, 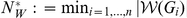, for a given population of 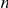 weighted networks denoted 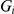. Thus, when considering sparser networks, such as structural brain networks, one may still be able to control for differences in cost, by integrating over a subset of the cost regimen, which reflects the sparsity of the networks under comparison.

A similar problem may arise if one or several networks in the population of interest have multiplicities, i.e. weights that take identical values. Since cost-integration relies on the ranking of weights, it follows that one may need to adjust for such multiplicities, otherwise this can lead to spurious generation of random topologies. That is, when the tied ranks are resolved by random ordering, the allocation of weights with identical values to specific cost levels is random, and therefore artificially create a random topology for these cost levels. For sparse networks, multiplicities are likely to arise around zero. However, for large non-sparse networks, the occurrence of multiplicities should be evaluated by counting the number of tied ranks in the distribution of the weights. In particular, if the two populations of networks that one wishes to compare differ significantly in number of tied ranks, then comparison based on cost-integration will be contaminated by an artificial level of random topology.

Another possible limitation of cost-integration is that by integrating over several cost levels, we omit to take into account the dependence between the topologies of the different thresholded graphs. The topological structure of the unweighted networks created by thresholding the original weighted graph share the same edges. Arguably, the cumulative nature of this procedure results in emphasizing the importance of the set of edges with the largest weights. Once these edges have been included into a thresholded graph, they will be retained for the remaining cost levels. This is especially true for the topological metrics that we have studied in this paper, since global and local efficiencies are both monotonic functions of cost.

### Extensions

Most of this paper has focused on the global efficiency metric. Thus, our conclusions and the examples studied will not necessarily apply to other topological measures. However, our main result (proposition 1) was proved in a very general setting, which is independent of the particular formula of the topological metric of interest. Our general conclusion about the usefulness of cost-integration when one wishes to disentangle differences in cost from differences in topology is therefore valid for any topological metric defined for an unweighted graph. In addition, we note that since most weighted metrics are constructed on the basis of the weighted shortest path matrix, one may surmise that our second main theoretical result (proposition 2), may hold in a more general setting. However, a proof that the equivalence relationship between the weighted version of a topological metric and the weighted cost, for instance, hold for topological measures other than the global efficiency would require further work.

Thus far, we have only considered user-defined distributions on the space of network costs. Future methodological developments will be needed in order to consider more sophisticated approaches to this problem. In particular, the specification of a probability mass function on 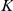 should take into account the effect size associated with different values of this random variable. When considering correlation coefficients, for instance, it can easily be shown that higher values indicate larger effects, and it may therefore be preferable to emphasize network comparisons built upon the largest correlation coefficients. This may be implemented by integrating network topological metrics with respect to a skewed distribution on 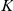, which puts more weight on sparse networks, whose edges are better identified.

One should note that the use of cost-integration when comparing weighted networks is not akin to taking into consideration the multilevel or hierarchical nature of a weighted network. Such a structural interpretation of the successive thresholding necessary for such an integration is not necessary to justify the usefulness of the method in controlling for monotonic differences in weighted cost. Since the networks of interest ‘exist’ as weighted networks, their thresholding remains artificial and it is not clear whether one can ascribe any substantive meaning to the resulting family of thresholded graphs. Further work will therefore be needed in order to better characterize the architecture of the ensemble of thresholded discrete networks subtending a weighted graph.

## Methods

### A: Monte Carlo (MC) Sampling

The cost-integrated quantities introduced in this paper may first appear unwieldy to compute, especially when considering large graphs. However, the structure of these integrals allows the construction of a straightforward MC sampling scheme. This classical approximation method has the advantage of providing both an estimate of the quantity of interest and an estimate of the variance of that estimation. For an introductory text to MC techniques, see Gilks et al. [40], and for a more advanced treatment, Robert et al. [41].

In order to apply MC sampling theory, we first observe that our integration problem –that is, the computation of 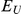
– can be re-formulated as an expectation. For convenience, we drop any reference to the function 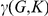, and therefore denote the efficiency metric 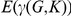 as 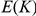. The uniformly cost-integrated metric 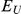 can then be expressed as an expectation of 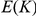 since, straightforwardly, we have
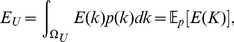
(41)where 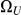 is the space of all possible costs for 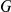, with 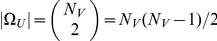. The expectation in (41) is taken with respect to 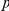, the probability mass function of 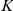, and explicit reference to 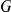 has been omitted. It is natural to consider the use of a sample 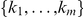 from 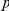 in order to approximate 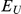 by the following empirical average,
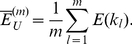
(42)The approximation 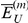 converges to 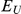 almost surely, by the Strong Law of Large Numbers. In addition, providing that 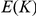 is square-integrable, the speed of convergence of 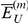 can be evaluated by considering the theoretical variance of that estimate,
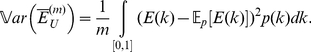
(43)which can be approximated by the following MC variance,
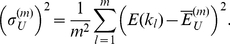
(44)This quantity is of special interest in MC sampling, as it permits the evaluation of the rate of convergence of the estimation. It is generally referred to as the *MC standard error*. Using Slutsky's theorem, it can also be shown that as 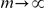, the random variable,
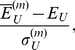
(45)has the probability density function of a Normal variate centered at zero, with unit variance. MC sampling is especially useful when the stochastic function that we wish to integrate –here, denoted 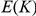
– is complex, whereas the random variable with respect to which we integrate can easily be sampled. Most topological metrics will be of a complex nature –i.e. non-linear. By contrast, the wiring cost 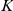 will be straightforward to sample, whether we specify a uniform or a Beta-binomial distribution on 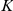. The theory underlying MC sampling is general and can therefore be applied to any type of topological metrics. Care, however, should be taken when evaluating the properties of very large networks, where the topology may vary substantially from one level of cost to another. When confronted with such large networks, the MC standard error remains a good indicator of the accuracy of such approximations.

### B: Proof of Proposition 1

In order to prove proposition (1), we first need to give a formal definition of 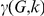, for some given weighted network 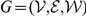. This function relies on the concept of rank, which can be formally defined in our context, as follows
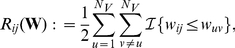
(46)where 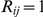 implies that 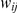 is the largest weight in 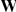. Here, we have assumed that there are no ties in the ranks of 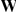. When ties occur in practice, we recommend to resolve tied ranks by assigning the corresponding ordering of the elements' indices. By contrast, resolving tied ranks using random allocation can result in introducing a spurious amount of random topology in the networks of interest. The presence of tied ranks, however, will generally be indicative of a high level of sparsity, which is better dealt with by restricting the domain of integration.

Computationally, this definition can be simplified if one only considers the upper off-diagonal elements of 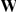 and omits the division by 2. For our purpose, this definition will be more convenient. These ranks can be standardized in order to derive the *percentile ranks*,
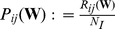
(47)where 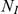 is the number of edges in the saturated version of 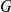. Note that the resulting matrices 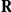 and 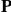 of ranks and percentile ranks, respectively, are both symmetric. A good introduction to order statistics, ranks and percentile ranks is provided by Lin et al. [42].

The function 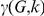 can now be given a formal definition using the 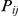
's, such that
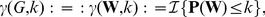
(48)where the indicator function is applied elementwise to matrix 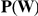, where 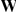 is the similarity matrix of 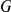. It can hence be seen that the function 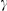 prescribes an adjacency matrix 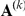 with the desired cost. This can be verified by computing the cost of the corresponding unweighted network 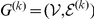, where 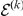 is the edge set that populates 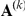, obtained after application of the 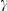 function at 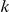. Provided that 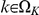, as defined in equation (16), we have
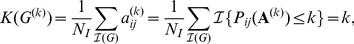
(49)which can be verified by noting that equation (49) is simply the discrete version of the integration of an indicator function of the form, 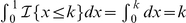. Using this notation, the proof of proposition 1 is now straightforward. This demonstration uses the fact that a monotonic function does not modify the ranks of its arguments.

*Proof.* Recall that the cost-integrated version of 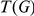, in its computational form, is given by
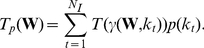
(50)To demonstrate that 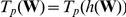, it therefore suffices to show that
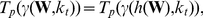
(51)for every 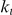, which further simplifies to the sole requirement that 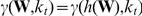, for all 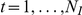. From the definition of the 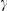 function introduced in equation (48), we have the following relationship,
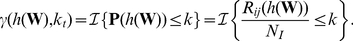
(52)However, one can observe that, since 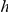 is applied elementwise, we have
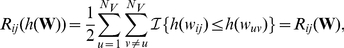
(53)for any monotonic function 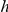. This completes the proof.

### C: Proof of Proposition 2

*Proof.* We prove the result by contradiction. Assume that the conclusion does not hold. That is, 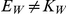. By applying the definitions of 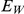 and 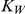 in equations (14) and (9), respectively, we have
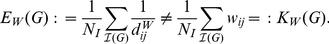
(54)It therefore suffices to show that 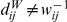 for at least one of the weights. The weighted shortest path 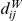 is defined in equation (12) as
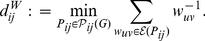
(55)It follows that 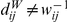 if and only if there exists a path 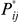 in 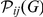, which satisfies
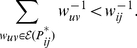
(56)That is, the path 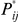 is shorter than the direct connection 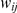 between the 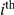 and 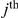 vertices. Inequality (56) can be sandwiched in the following fashion,
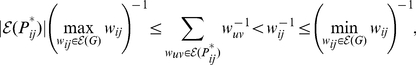
(57)where 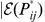 denotes the cardinality of 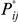. Inverting the entire inequality then gives
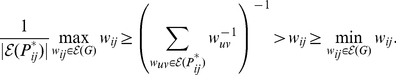
(58)However, we clearly have
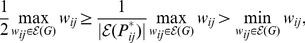
(59)which contradicts our hypothesis, and proves the claim.
